# Joint Final Report of EORTC 26951 and RTOG 9402: Phase III Trials With Procarbazine, Lomustine, and Vincristine Chemotherapy for Anaplastic Oligodendroglial Tumors

**DOI:** 10.1200/JCO.21.02543

**Published:** 2022-06-22

**Authors:** Andrew B. Lassman, Khê Hoang-Xuan, Mei-Yin C. Polley, Alba A. Brandes, J. Gregory Cairncross, Johan M. Kros, Lynn S. Ashby, Martin J.B. Taphoorn, Luis Souhami, Winand N.M. Dinjens, Nadia N. Laack, Mathilde C.M. Kouwenhoven, Karen L. Fink, Pim J. French, David R. Macdonald, Denis Lacombe, Minhee Won, Thierry Gorlia, Minesh P. Mehta, Martin J. van den Bent

**Affiliations:** ^1^ Division of Neuro-Oncology, Department of Neurology, Columbia University Vagelos College of Physicians and Surgeons, New York, NY; ^2^ Herbert Irving Comprehensive Cancer Center, New York, NY; ^3^ NewYork-Presbyterian Hospital, New York, NY; ^4^ AP-HP, Sorbonne Université, Hôpitaux Universitaires La Pitié Salpêtrière - Charles Foix, Service de Neurologie 2, Paris, France; ^5^ NRG Oncology Statistics and Data Management Center, Philadelphia, PA; ^6^ Department of Medical Oncology, AUSL/IRCCS Institute of Neurological Sciences, Bologna, Italy; ^7^ Charbonneau Cancer Institute, University of Calgary, Calgary, Canada; ^8^ Department of Pathology, Erasmus MC Cancer Institute, University Medical Center, Rotterdam, the Netherlands; ^9^ Barrow Neurological Institute, Phoenix, AZ; ^10^ Department of Neurology, Leiden University Medical Center, Leiden, the Netherlands; ^11^ Department of Neurology, Haaglanden Medical Center, the Hague, the Netherlands; ^12^ Department of Radiation Oncology, McGill University, Montreal, Quebec, Canada; ^13^ Mayo Clinic Accruals for Rochester Methodist Hospital, Rochester, MN; ^14^ Department of Neurology, Amsterdam Universities Medical Centers, location VUmc, Amsterdam, the Netherlands; ^15^ Baylor University Medical Center, Houston, TX; ^16^ Department of Neurology, Erasmus MC Cancer Institute, Rotterdam, the Netherlands; ^17^ London Regional Cancer Program, Western University, London, Canada (RT); ^18^ EORTC, Brussels, Belgium; ^19^ Miami Cancer Institute, Miami, FL

## Abstract

*Clinical trials frequently include multiple end points that mature at different times. The initial report, typically based on the basis of the primary end point, may be published when key planned co-primary or secondary analyses are not yet available. Clinical Trial Updates provide an opportunity to disseminate additional results from studies, published in* JCO *or elsewhere, for which the primary end point has already been reported.*

Anaplastic oligodendroglial tumors (AOTs) are chemotherapy-sensitive brain tumors. We report the final very long-term survival results from European Organization for the Research and Treatment of Cancer 26951 and Radiation Therapy Oncology Group 9402 phase III trials initiated in 1990s, which both studied radiotherapy with/without neo/adjuvant procarbazine, lomustine, and vincristine (PCV) for newly diagnosed anaplastic oligodendroglial tumors. The median follow-up duration in both was 18-19 years. For European Organization for the Research and Treatment of Cancer 26951, median, 14-year, and probable 20-year overall survival rates without versus with PCV were 2.6 years, 13.4%, and 10.1% versus 3.5 years, 25.1%, and 16.8% (N = 368 overall; hazard ratio [HR] 0.78; 95% CI, 0.63 to 0.98; *P* = .033), with 1p19q codeletion 9.3 years, 26.2%, and 13.6% versus 14.2 years, 51.0%, and 37.1% (n = 80; HR 0.60; 95% CI, 0.35 to 1.03; *P* = .063), respectively. For Radiation Therapy Oncology Group 9402, analogous results were 4.8 years, 16.5%, and 11.2% versus 4.8 years, 29.1%, and 24.6% (N = 289 overall; HR 0.79; 95% CI, 0.61 to 1.03; *P* = .08), with codeletion 7.3 years, 25.0%, and 14.9% versus 13.2 years, 46.1%, and 37% (n = 125; HR 0.61; 95% CI, 0.40 to 0.94; *P* = .02), respectively. With that, the studies show similar long-term survival even without tumor recurrence in a significant proportion of patients after first-line treatment with radiotherapy/PCV.

## INTRODUCTION

European Organization for Research and Treatment of Cancer (EORTC) and Radiation Therapy Oncology Group (RTOG, now NRG Oncology) developed open-label randomized phase III trials (EORTC 26951 and RTOG 9402) in the 1990s testing the addition of procarbazine, lomustine, (CCNU) and vincristine (PCV) chemotherapy to radiotherapy (RT) for newly diagnosed anaplastic oligodendroglial tumors. Both studies reported initially that adding PCV improved progression-free survival (PFS) but not overall survival (OS).^1,2^ However, with longer follow-up, improved OS was also observed, particularly in patients with chromosome 1p/19q codeleted tumors.^3,4^ The importance of 1p/19q codeletion,^5^ isocitrate dehydrogenase (*IDH*) 1 and 2 mutations,^6^ and O^6^-methylguanine-methyltransferase promoter methylation^7^ in tumor DNA was not established when these trials were launched. However, both trials centrally analyzed available tumor tissue retrospectively.^3,4^ Now, nearly 30 years since EORTC 26951 and RTOG 9402 were launched, we report extremely long-term follow-up of fully mature and final survival data from both trials and results in molecular subgroups in line with the current WHO Classification of Tumors of the Central Nervous System.^8^

**CONTEXT**

**Key Objective**
The very long-term survival of patients with anaplastic oligodendroglioma who received (neo)adjuvant procarbazine, lomustine, and vincristine chemotherapy in addition to radiotherapy as part of the initial treatment is unknown.
**Knowledge Generated**
In two independent prospective randomized trials, the benefit of (neo)adjuvant procarbazine, lomustine, and vincristine added to radiotherapy in anaplastic oligodendroglioma was demonstrated. With an estimated survival after 20 years in the 35% range, a sizeable proportion of patients with a 1p/19q codeleted oligodendroglioma (WHO classification 2021) achieve long-term survival.
**Relevance**
Long-term survival is possible in a significant proportion of patients with a 1p/19q codeleted oligodendroglioma, even in patients with histologically anaplastic tumors, emphasizing the importance of quality of survival and need for adequate supportive measures.

## METHODS

Entry criteria, trial design, stratification factors, and statistical methods were described previously and are detailed in the Data Supplement (online only). Briefly, RT was administered to a total dose of 59.4 Gy in 33 fractions of 1.8 Gy each in both studies. In EORTC 26951, patients received up to six cycles of adjuvant PCV after RT; in RTOG 9402, patients received up to four cycles of intensified PCV before RT.^9^ Molecular analyses were performed centrally post hoc. PFS was defined as the time from random assignment to disease progression or death from any cause, whichever occurred first. Progression was defined locally using the Macdonald criteria.^10^ OS was defined as the time between random assignment and death due to any cause. Duration of follow-up was assessed with the inverse Kaplan-Meier method.

## RESULTS

### EORTC 26951

**FIG 1.**
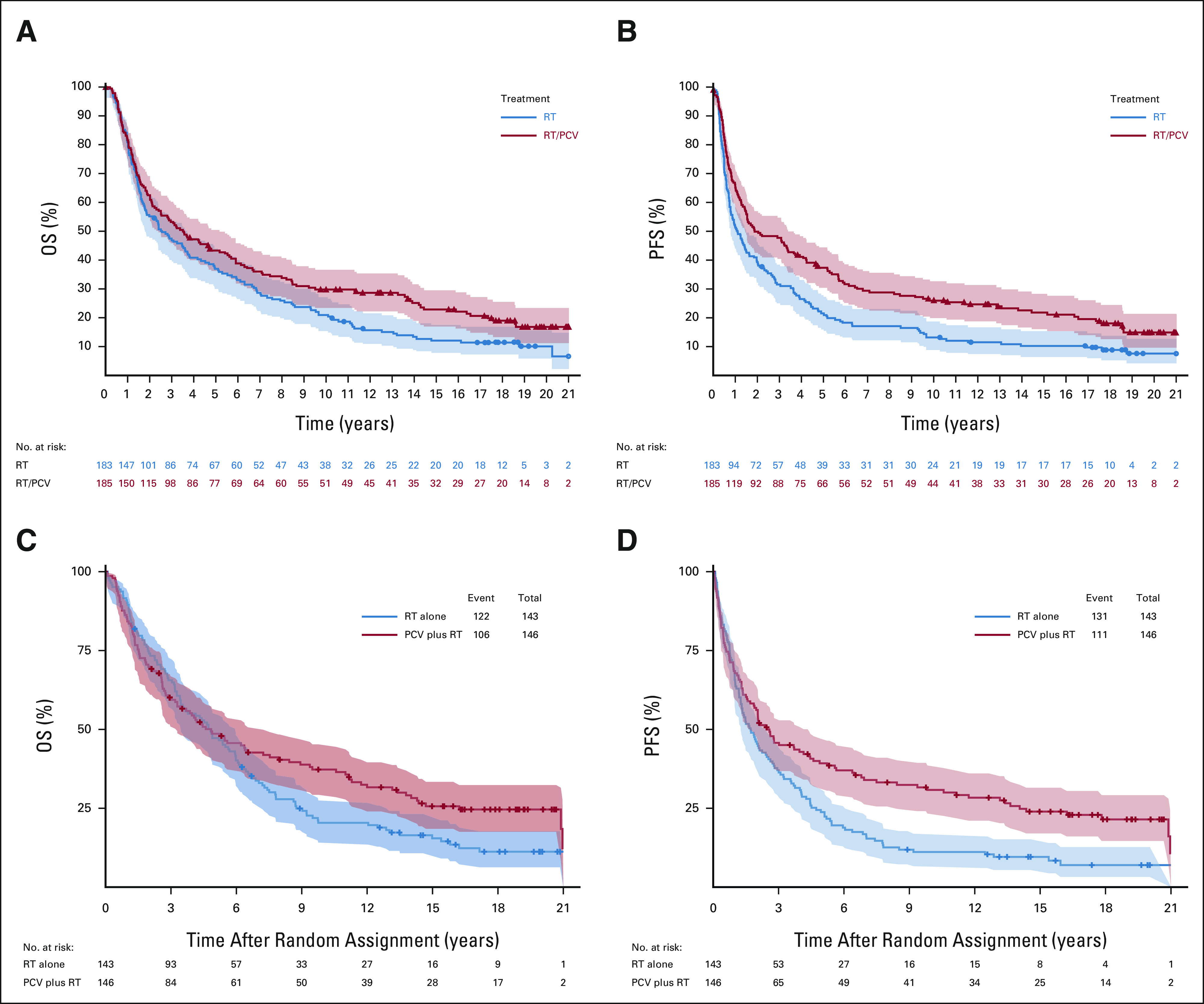
OS and PFS in the intent-to-treat populations. (A) OS by treatment arm in EORTC 26951, intent-to-treat population. (B) PFS by treatment arm in EORTC 26951, intent-to-treat population. (C) OS by treatment arm in RTOG 9402, intent-to-treat population. (D) PFS by treatment arm in RTOG 9402, intent-to-treat population. EORTC, European Organization for the Research and Treatment of Cancer; OS, overall survival; PCV, procarbazine, lomustine, and vincristine; PFS, progression-free survival; RT, radiotherapy; RTOG, Radiation Therapy Oncology Group.

**FIG 2.**
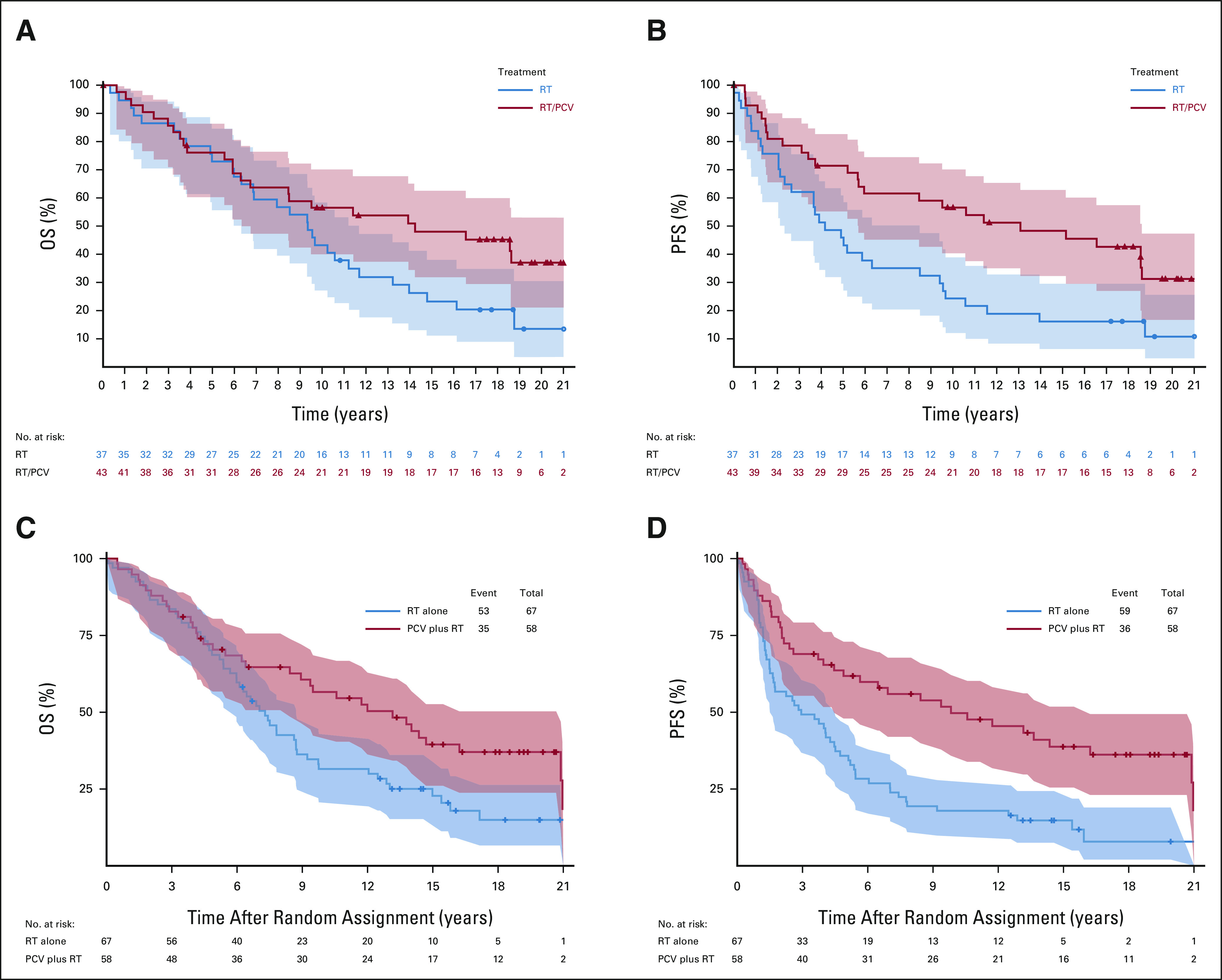
OS and PFS in the patients with 1p/19q codeleted tumors. (A) OS by treatment arm among 1p/19q codeleted cases in EORTC 26951, assessed with FISH. (B) PFS by treatment among 1p/19q codeleted cases in EORTC 26951 (FISH). (C) OS by treatment arm among 1p/19q codeleted cases in RTOG 9402. (D) PFS by treatment arm among 1p/19q codeleted cases in RTOG 9402. EORTC, European Organization for the Research and Treatment of Cancer; FISH, fluorescent in situ hybridization; OS, overall survival; PCV, procarbazine, lomustine, and vincristine; PFS, progression-free survival; RT, radiotherapy; RTOG, Radiation Therapy Oncology Group.

Between August 1996 and March 2002, 368 patients were enrolled. The database was locked for analysis on June 21, 2019 with 61 of the randomly assigned 368 patients (17%) still alive. For all patients, the median follow-up for PFS and OS is 19 years. The median follow-up of surviving patients free of progression is 17.7 years (range > 0-21.3 years). The median follow-up of surviving patients is 17.8 years with range (> 0-21.7 years). For EORTC 26951, codeletion of 1p/19q by fluorescent in situ hybridization (FISH) was observed in 25% (80 of 316),^3^ and *IDH*-1 or -2 mutation was detected by Sanger sequencing in 46% (83 of 182) of informative cases. Most (39 of 45, 87%) tumors classified as codeleted by FISH were *IDH*-mutant. One hundred fifteen (115) tumors were analyzed with genome-wide methylation arrays.^11-13^ Finally, 139 tumors were assayed by next-generation sequencing (using Ion Torrent).^14^ Table 1 and the Data Supplement, Table 1C summarizes the PFS and OS in the intent-to-treat population and the various molecularly defined subgroups from both studies. Both PFS and OS increased with the addition of PCV to RT, PFS: hazard ratio [HR] 0.69; 95% CI, 0.55 to 0.86; *P* = .001; OS: HR 0.78; 95% CI, 0.63 to 0.98; *P* = .033 (Figs 1A and 1B). In the 80 patients with 1p/19q codeleted tumors, 26 (33%) were still alive at the time of last follow-up. In this subgroup, the HR for PFS was 0.49; 95% CI, 0.29 to 0.83; *P* = .007 and for OS 0.60; 95% CI, 0.35 to 1.03; *P* = .063 (Figs 2A and 2B). In the 43 patients with IDH-mutant tumors but no 1p/19q codeletion, the HR for PFS was 0.57; 95% CI, 0.29 to 1.10; *P* = .091 and for OS 0.60; 95% CI, 0.31 to 1.17; *P* = .131 (Table 1, see also the Data Supplement). Detailed prognostic and predictive makers analyses are presented in the Data Supplement. Only *methylguanine-methyltransferase* promoter methylation as assessed with methylation arrays proved to be significantly predictive of benefit from PCV (HR 0.41; 95% CI, 0.25 to 0.67; *P* < .0001).

**TABLE 1.**
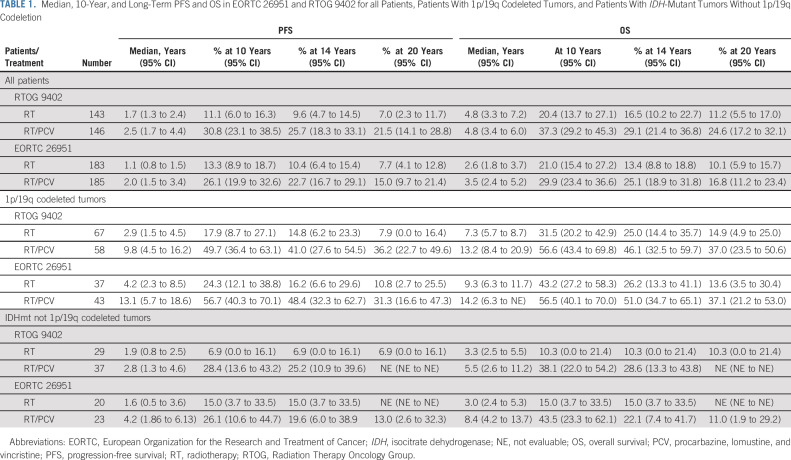
Median, 10-Year, and Long-Term PFS and OS in EORTC 26951 and RTOG 9402 for all Patients, Patients With 1p/19q Codeleted Tumors, and Patients With *IDH*-Mutant Tumors Without 1p/19q Codeletion

### RTOG 9402

Between July 1994 and March 2002, 289 patients were randomly assigned. The database was locked for this analyses on May 21, 2018 with 61 patients still alive (21%) and 47 (16%) free of progression. The median follow-up of surviving patients free of progression is 17.4 years (range > 0-23.1 years). The median follow-up of surviving patients is 18.1 years (range > 0-23.1 years). For RTOG 9402, codeletion was observed in 48% (125 of 261; FISH), and *IDH* mutation was detected (immunohistochemistry or DNA sequencing) in 74% (156 of 210; 154 in *IDH1* and two in *IDH2*) of informative cases. 1p/19q codeletion was observed mainly (90%) in *IDH*-mutant tumors.^15^ In the intent-to-treat analysis, the addition of PCV to RT improved PFS (HR = 0.67; 95% CI, 0.52 to 0.86) but not OS (HR 0.79; 95% CI, 0.61 to 1.03; Table 1 and Data Supplement, Table 1C; Figs 1C and 1D). Among patients with 1p/19q codeleted tumors (n = 125), receipt of PCV was associated with longer PFS and OS (PFS: HR 0.46; 95% CI, 0.30 to 0.70; *P* < .001; OS HR 0.61; 95% CI, 0.40 to 0.94; *P* = .02; Figs 2C and 2D). Among patients with non-codeleted *IDH*-mutant tumors (n = 66), PFS was significantly longer after PCV and RT than RT alone (HR 0.58; *P* = .046), with a trend toward longer OS with PCV HR 0.60; 95% CI, 0.34 to 1.03; *P* = .06 (Table 1, see also the Data Supplement). Detailed prognostic and predictive makers analyses are presented in the Data Supplement.

## DISCUSSION

EORTC 26951 and RTOG 9402 were practice-changing phase III trials. Here, we report very long-term mature and final survival analyses nearly 30 years after the trials were conceived. Both trials showed that the addition of PCV chemotherapy to RT lengthens disease control and survival relative to RT alone as first-line therapy for anaplastic oligodendroglial tumors, particularly among 1p/19q codeleted cases. We confirm the 40% reduction in the risk of death in both trials from adding PCV to RT in patients with 1p19q codeleted tumors, with a median survival after random assignment of approximately 14 years and estimated PFS and OS probabilities at 20 years from random assignment of 30% and 35%, respectively. Therefore, durable disease control and long survival are possible after PCV-based chemoradiotherapy as first-line treatment for patients with anaplastic oligodendroglioma. PCV chemoradiotherapy also improves survival for patients with *IDH*-mutant tumors without codeletion, although less robustly, which is consistent with results from RTOG 9802 for low-grade gliomas and with temozolomide in EORTC 26053-22054 (CATNON) for anaplastic gliomas.^16-18^ Our data show the long-term outcome in these patients with molecularly classified gliomas and reinforce the importance of PCV as a therapeutic chemotherapy regimen for gliomas with *IDH* mutation and especially 1p/19q codeletion, despite the transition by many to temozolomide as the drug of choice because of lower toxicity and perceived equivalence of efficacy.^19-25^ The long-term survival observed also emphasizes the need to better understand the long-term adverse effects of treatment, such as cognitive function and ability to continue living independently many years after treatment. The risk of late neurocognitive injury from early RT combined with efficacy of PCV prompted some investigators to defer RT altogether for codeleted cases (ClinicalTrials.gov identifier: NCT02444000 from which results are eagerly awaited). A concern, however, is that initial treatment with chemotherapy alone may be detrimental for survival.^25-27^ Finally, our ability to conduct and report extremely long-term survival results demonstrates the critical importance of governmentally funded networks in conducting clinical trials, particularly for indolent tumors with long follow-up required for full maturity, that are impossible for a commercial sponsor.
